# Multiview motion tracking based on a cartesian robot to monitor *Caenorhabditis elegans* in standard Petri dishes

**DOI:** 10.1038/s41598-022-05823-6

**Published:** 2022-02-02

**Authors:** Joan Carles Puchalt, Jose F. Gonzalez-Rojo, Ana Pilar Gómez-Escribano, Rafael P. Vázquez-Manrique, Antonio-José Sánchez-Salmerón

**Affiliations:** 1 Instituto de Automàtica e Informàtica Industrial, Universitat Politècnica de València, Valencia, Spain; 2 Laboratory of Molecular, Cellular and Genomic Biomedicine, Instituto de Investigación Sanitaria La Fe, Valencia, Spain; 3 Centro de Investigación Biomédica en Red de Enfermedades Raras (CIBERER), Valencia, Spain

**Keywords:** Optical sensors, Optical metrology, Imaging and sensing

## Abstract

Data from manual healthspan assays of the nematode *Caenorhabditis elegans* (*C. elegans*) can be complex to quantify. The first attempts to quantify motor performance were done manually, using the so-called thrashing or body bends assay. Some laboratories have automated these approaches using methods that help substantially to quantify these characteristic movements in small well plates. Even so, it is sometimes difficult to find differences in motor behaviour between strains, and/or between treated vs untreated worms. For this reason, we present here a new automated method that increases the resolution flexibility, in order to capture more movement details in large standard Petri dishes, in such way that those movements are less restricted. This method is based on a Cartesian robot, which enables high-resolution images capture in standard Petri dishes. Several cameras mounted strategically on the robot and working with different fields of view, capture the required *C. elegans* visual information. We have performed a locomotion-based healthspan experiment with several mutant strains, and we have been able to detect statistically significant differences between two strains that show very similar movement patterns.

## Introduction

*Caenorhabditis elegans* is a 1-mm-long worm that is widely used as a model animal in biology, but also in preclinical assays to test drugs, which may be used later in higher organisms before progressing to regular clinical studies^1^. Some trials consist of treating the worms with drugs, in parallel with the appropriate controls, and then subjecting them to lifespan or healthspan assays^2^. The simplest experiment to quantify is lifespan^1,3^, which studies the conditions that induce and increase the lifespan of a given organism. Nevertheless, longevity is not the only factor to consider in human health, but also how healthy the individual is for the entire time period. Thus, there are experiments to quantify healthspan which asses life quality.

Healthspan is generally described as the period in life during which the organism is in good health. In human clinical settings, grip strength, gait analysis and ability to perform daily tasks are often used as criteria to assess good health. In *C. elegans*, several physiological and functional parameters that change with age can be studied, such as lipofuscin accumulation or pharyngeal pumping^4^. Among these, the most powerful predictor of longevity appears to be movement. As in humans, *C. elegans*’ ability to move diminishes with aging, as they decline towards a state of frailty where they are only able to move their head, characteristic of the later stages in life.

In *C. elegans*, healthspan can be measured by analysing motility behaviour^5^. Researchers understand the amount of movement, coordination, body bends, reversals, pirouettes, travel distances or orientations can determine if there is neuronal, muscular or sensory damage, as well as animal proactivity. Some of the traditional methods to quantify movement is to check the number of (1) thrashing in a time period^6^, or (2) the maximum distance that the worm moves, from a given point, among others. In our case, we are comparing worms suffering from locomotor problems due to neurodegenerative diseases, with healthy worms (N2). Therefore, only a movement parameter (such as thrashing) can be used to assess health^7^.

Using manual methods, it is difficult to quantify movement, because it is not easy to visually measure a displacement or a trajectory angular variation. As stated above, quantification of movement enables us to study the conditioning factors altering health, whether these are drug treatments, different food intake or inter-strain genetic differences. These assays can identify harmful conditions (i.e. unhealthy food, drugs, deleterious mutations, etc.), so they can be neutralized. Conversely, they can be promoted, if they are beneficial. Importantly, the differences in behaviour between conditions and/or strains can be very subtle, and the statistical tests are not able to point out these variances. Therefore, it is important to introduce new methods to analyse these behaviours in greater detail. In summary, there are several drawbacks in manual experiments that make automated experimentation essential.

Some of the automated systems rely on taking image sequences of the whole Petri plate area^8–11^, which cannot capture high-resolution images of *C. elegans*. Most of healthspan automated systems require restricted worm movement (e.g. using glue or microchambers) thus allowing high-resolution image capture with a fixed camera. There are systems in which this restriction is performed with medium-sized multiwell plates^12^ harbouring a few nematodes. Also, small-sized multiwell plates are used^4,13–15^ in which just one animal is placed, usually to control individual identity in a healthspan study throughout its lifespan^16^. These small wells are almost worm size, and due to this small restricted area, the movement variations can be captured by a camera at high-resolution. However, under these restrictions, the nematodes cannot perform certain movements, trajectories and interrelations. Another method that has many advantages involves the use of microfluidic chambers to restrict movements, but again carries the same problems^17–20^. On the other hand, there are also micro-pillars^21–24^, which force the worms to pass through predetermined paths, which also alter the trajectories.

Our main objective is to obtain high-resolution images in large Petri dishes, for which is necessary to locate any worm on low-resolution image and then find and tracking them with high-resolution image. The hypothesis is by processing high-resolution image we could find significant statistical differences between the movement of several strains. Our method, described herein, allows software-processed tracking of all worms, maintaining their individual identity at low-resolution, while mechanical tracking can be done using another camera with higher resolution. In this way, all worms can be assessed individually at two different resolutions, in a less restricted setting, with longer and more coordinated movements. This allows for social interactions of multiple worms in a less artificial environment. Here, we describe this new method as a proof-of-concept, and we compare automated versus manual assays, in order to assess whether both techniques provide similar results. Our experiments demonstrated significant statistical differences in movement between the wild type standard (N2 Bristol), a strain expressing a long track of CAG triplets (RVM66) and *unc-1* mutants, which show strong non-coordination, hence validating this method.

## Materials and methods

**Figure 1:**
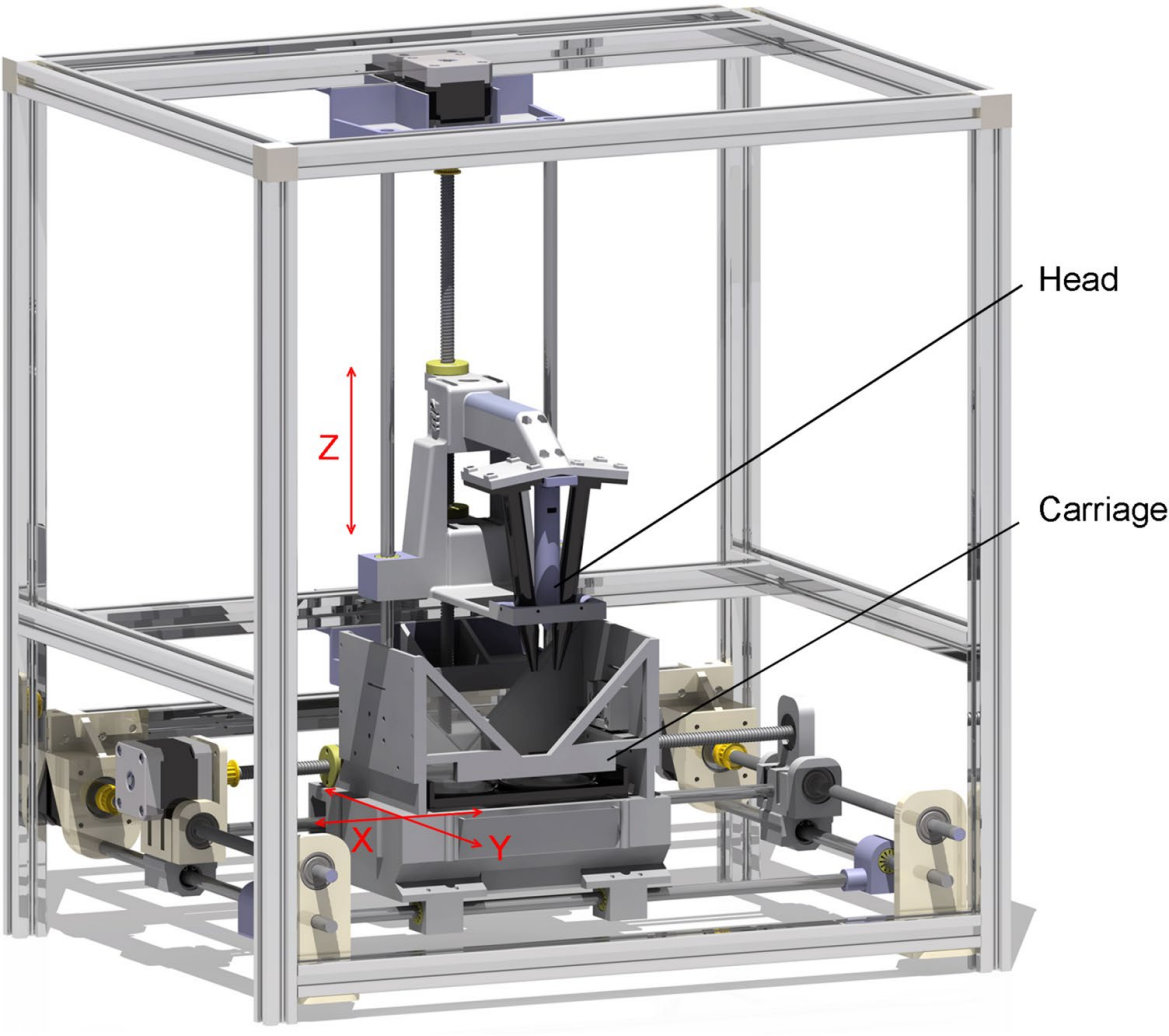
Multi-view cartesian robot. The image was obtained with SOLIDWORKS 2020 SP3.0. This is the general view, showing all components.

Before going into more detail, at this point, we give a general description of the method presented in the current manuscript: It is based on a Cartesian robot (Fig. 1), which moves a carriage on the XY axes. On the Z axis, a head axially moves up and down, this head holds two high-resolution cameras (named microcameras) which have focal length of 102.61 mm and 1.09 $\upmu$/pxl and a laser to locate both microcameras. The carriage has a backlight, a Petri plate support for two plates, a beam splitter and a low-resolution camera (named macrocamera) with focal length of 3.6 and $27.31 \upmu$/pxl per plate. In this way, we have two different resolutions at the same time to inspect the same Petri dish. Detailed techniques to achieve this concept are described in the following points.

The device structure has been assembled with $20 \times 20$ mm aluminium profiles. The parts have been designed to hold the motors, shafts and PCBs in the structure. These clamping parts, as well as the head and the carriage, have been printed with PLA material in a 3D printer (BCN3D Sigma 3D printer-6). Four Nema 17 stepper motors have been employed, a Teensy 3.6 microcontroller has been used to control them and a DRV8825 to feed each motor. Regarding the image capture, it consists of four Raspberry Pi v.4 with cameras (picam v1.3), configured 2592 $\times$ 1944@3Hz. The lighting system is a 7′′ 800 $\times$ 480 dot display controlled by one Raspberry Pi. To achieve the light beam reflection/transmission, a 45 and 50T/50R beamsplitter is used, thus the reflected light has a 45 inclination while the transmitted light is not deformed. Finally, the Master is an Intel Desktop Computer i7-4790 CPU@3.60GHzx8, 7.6 GiB RAM and Intel Haswell Desktop Graphics.

### Vision system

The vision system is composed of two microcameras and two macrocameras, which inspect two 55 mm diameter Petri plates. Each macrocamera captures an entire plate, while the microcameras magnify a small region of interest where a worm is located.

#### The carriage

**Figure 2:**
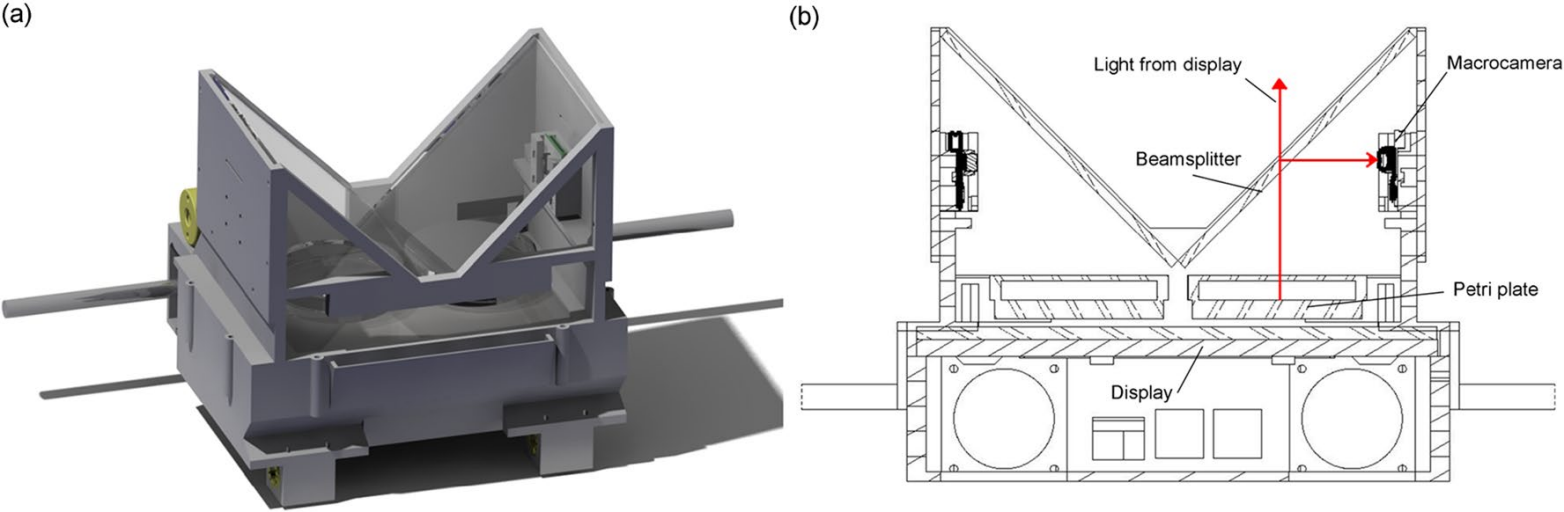
The XY carriage. The image was obtained with SOLIDWORKS 2020 SP3.0. (**a**) The whole carriage, (**b**) a section view to see parts in detail.

The carriage (Fig. 2a,b) has the backlight (display), the Petri plate support, the beam splitters and the macrocameras assembled on itself. The lighting is a 7′′ display, on which a Petri plate support is fixed. Each beamsplitter is placed fulfilling two restrictions, one is it must keep $45^{\circ }$ with respect to the surface of the display and therefore with respect to its illumination. Another restriction is that the beamsplitter must be large enough to reflect and transmit the entire Petri plate image. Each macrocamera is positioned perpendicular to the display and at a position and distance such that the reflected image from the display remains centred and complete in the camera. In other words, the Petri plate image remains fixed with respect to the macrocamera, despite the fact that the carriage moves in X and Y, thereby all the worms in the plate can be tracked in the low-resolution images sequence uninterruptedly. The display is also fixed with respect to the macrocamera, therefore each point drawn on the display can be correlated with a camera pixel, this allows active vision^25^.

#### Active vision

Active vision is used to control lighting, thereby the intensity and light colour that each worm receives can be regulated by the following methodology: the macrocamera and the display form a closed control loop, which makes all the pixels in the image reach the same reference. See^25^ to know how to calibrate the camera with respect to the display, in order to find the transformation matrix correlating each texel display with each camera pixel (in this case the macrocamera). When there is a Petri dish between display and camera, the light received by the camera has different grey level due to worm shadows. The controller will illuminate more intensely the texels associated to those darker image pixels than the reference detected; and when pixels are brighter than the reference, then the intensity will be reduced to the associated texels.

#### The head

**Figure 3:**
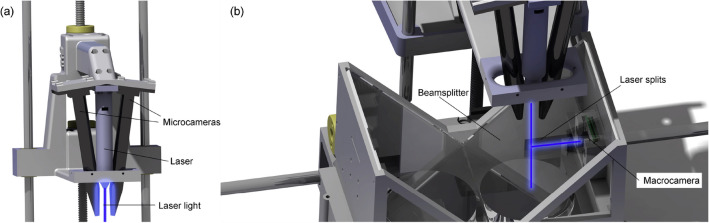
The head. The image was obtained with SOLIDWORKS 2020 SP3.0. (**a**) The head has two microcameras and one laser. (**b**) The head and de carriage are assembled, where the laser light trajectory is shown.

The head (Fig. 3a,b) has both microcameras and the laser, to know the head position with respect to the carriage, assembled thereon. It has two cameras, thus enabling stereovision. The cameras have a certain angle ($8^{\circ }$) to be able to take various perspectives, which makes it possible to orientate both cameras to capture the same region of interest (where the worm is). And, on the other hand, this angle causes a necessary space between the lenses, where a laser can be set, whose light falls orthogonally on the Petri dish surface. The laser projects a point on the macroimage, which gives the head position (microcameras) with respect to the macrocamera. In this way, the carriage position can be corrected, and thus microcameras can capture a desired worm-target selected from global information captured by the macrocamera. The microcameras are adjusted in such a way that the laser pointer is right in the centre of each image, thus when the laser pointer is in the target worm position, this worm will be displayed at both image centres.

### Control system

The algorithms have been developed in C++, with the Robotic Operating System (ROS) which is a tool for distributed systems and the computer vision library is OpenCV. The Operating System is Ubuntu, an Ubiquity distribution runs on the Raspberry Pi4 that has the ROS installation. The Master is launched on a Desktop Computer where UBUNTU 18.04.5 LTS and ROS melodic are installed and running.

**Figure 4:**
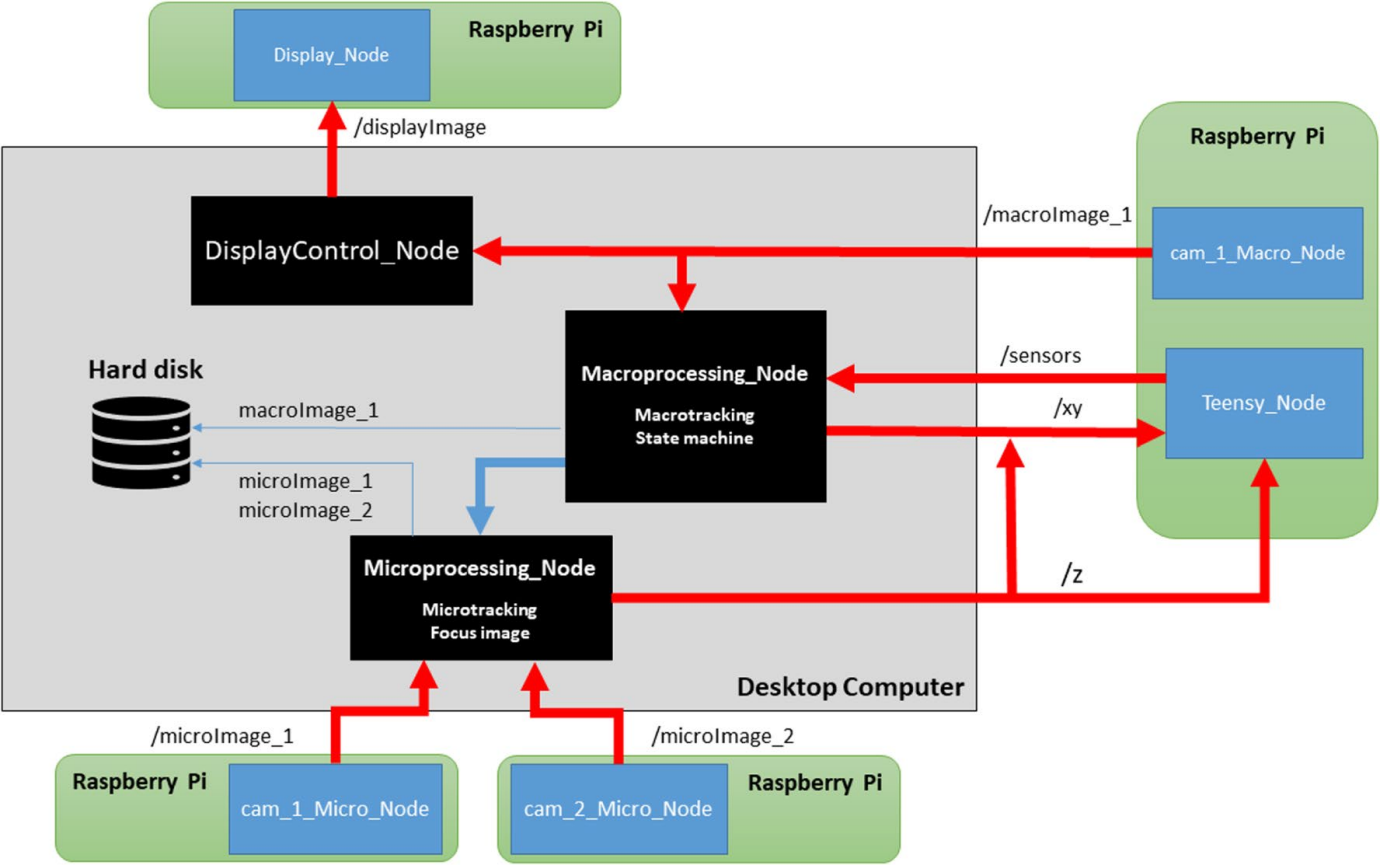
ROS diagram. Black squares are ROS nodes running on desktop computer, green squares are the four Raspberries Pi and blue squares are ROS nodes running on Raspberries Pi. Macroprocessing_Node implements the macroimage processing, macrotracking and state machine. Microimage_Node implements both microimage processing, microtracking and microimage focus (by changing Z axe). The nodes: cam_1_Micro_Node, cam_2_Micro_Node and cam_1_Macro_Node, execute the camera driver, take the image and publish the images into /microImage_1, /microImage_2 and /macroImage_1 respectively.

The ROS structure is shown in Fig. 4. The Master has both nodes of image processing and control. At least the same number of Raspberries Pi as cameras are required, so each camera will have its ROS node running on a Raspberry Pi, which will publish the image. One Raspberry has a ROS node, which connects via serial to a Teensy 3.6, this node subscribes to a topic where the movement steps are published by another node (Macroprocessing_Node and/or Microprocessing_Node). And another Raspberry Pi launches other node that executes the display driver and subscribes to the display image.

Finally, the processing and control nodes are launched in the Master:

- DisplayControl_Node is the one that controls the display lighting. This is subscribed to the macroimage topic, it performs the macrocamera calibration, the image transformation, compensates the lighting to obtain the reference level and publishes the lighting pattern calculated into /displayImage, thereby its subscriber (Display_Node) can draw the illumination pattern on the display.
- Macroprocessing_Node subscribes to the macroimage topic (macrocamera). This node processes the image, locates the targets, makes the framing on worm selected with macroimage information and contains a *state machine*, which sequences the tasks. The image processing consists of three steps; (1) an adaptative threshold is used for image segmentation; (2) the objects are classified by size; and (3) the object identification is tracked by the minimum distance between the last object locations to the current locations. Further details of the image processing are shown in Supplementary Fig. 1.
- Microprocessing_Node, subscribed to the both microcameras, does the image processing, focusing the image via publishing Z variable and also performs the microtracking by publishing on XY variable, which is made with microimage information. Further details of the image processing are shown in Supplementary Fig. 2.

**Figure 5:**
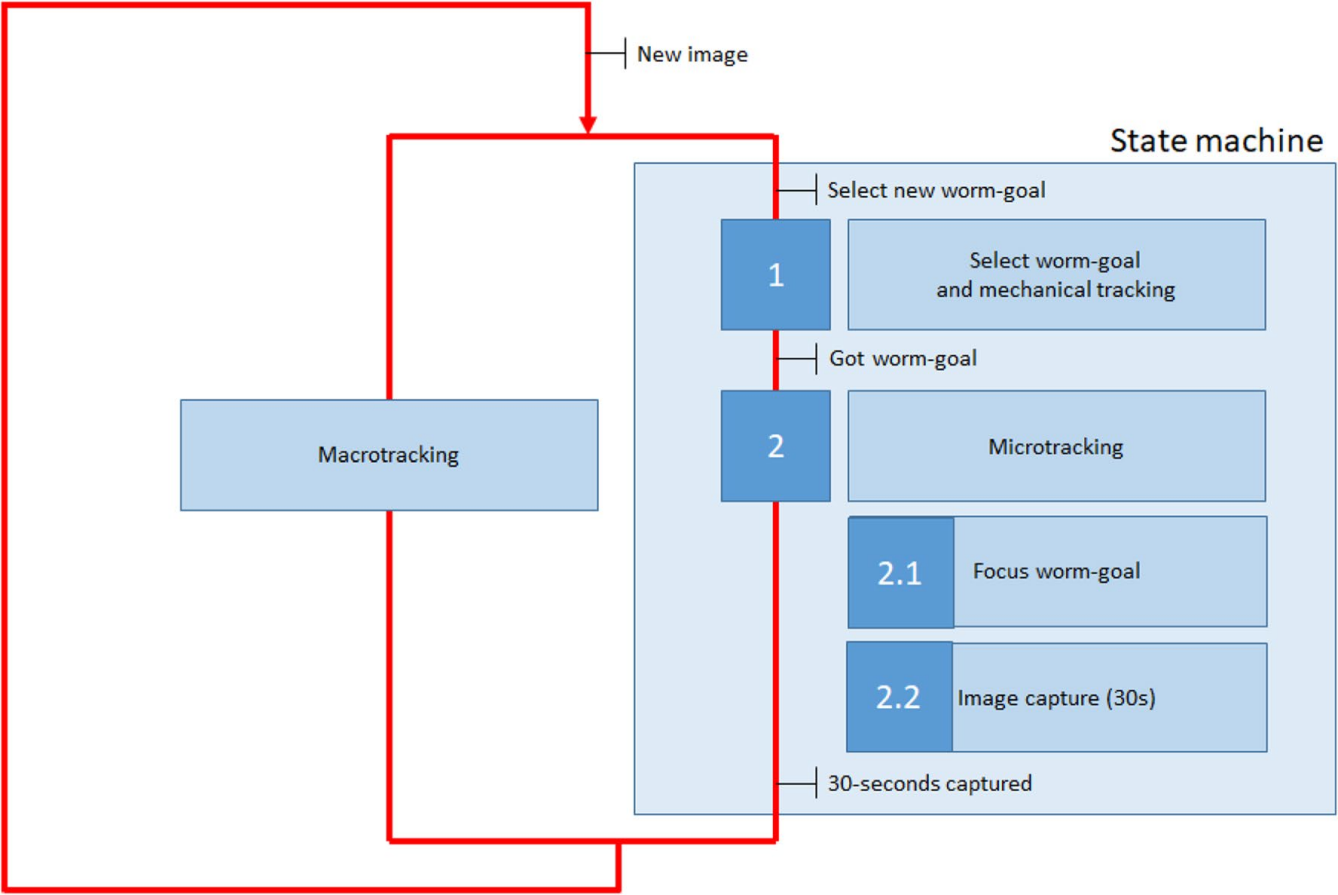
State machine. This flowchart shows the tasks of the state machine. Macrotracking is running constantly (it is software tracking), while state machine executes (1) when the worm has been selected. The 2-state starts when worm-goal has been achieved (the worm selected is framed). And when a 30-s sequence has been captured, then the mechanical tracking ends and the loop restarts.

The *state machine* (Fig. 5) sequences all the tasks: all worms constant macrotracking (1) worm selection and worm-goal acquisition (2) selected worm microtracking (2.1) selected worm focus in microcameras (2.2) both microimage capture.

The distance between the plate and the macrocamera is fixed, thus the relationship between the pixels and the motor steps can be known, by calculating the needed step number to move a certain pixel number. This model may have a small variability due to the agar height variability, 1 mm in Z (height) produces an error of 0.338 mm in X and Y, as shown in Eq. (1), where the half of view field is $\alpha =41.41^{\circ }/2=20.7^{\circ }$. With this small error, this model is a good approximation, which can be corrected with a proportional regulator.

1 $$\begin{aligned} \Delta x=\Delta z \cdot \tan {\alpha }. \end{aligned}$$

Tracking is carried out at two levels (1) macrotracking and (2) microtracking. Macrotracking constantly locates all the worms on the plate and identifies them using software, providing data on which worms have been tested and which have not (Supplementary Video 1). Among all worms, one is selected (worm selection state), whose centroid in pixels is known just as the laser position. The error is calculated and multiplied by the *pixel*/*step* ratio, the result is the necessary step number to reach the worm position, which is sent to the motor controllers. In one or two iterations the target is reached and when the error is small ($e < 15 {\text{pxl}}$) the state is switched to microtracking. For microtracking, the laser is unnecessary, because the worm occupies a large image part and remains centred on it. What is more, the laser also impoverishes the image quality because it emits another light component besides backlight; furthermore, it saturates a small spot on the microimage. For these reasons, the designed sequence is as follows: the laser is turned off, the worm centroid is obtained and it is carried to its centre as the nematode moves. While microtracking is performed, two sequentially subroutines are executed: first the nematodes are placed in focus and, second, the microimages are captured at 2 fps for 30 s (in our case). For this other tracking we also calculate a model to approximately determine the *pixel*/*step* ratio (1.14).

### Experiment design

We prepared three conditions with three different mutant strains, the wild type (N2 Bristol) strain is the control condition, the study condition is RVM66, and as a negative control with a mutant with obvious mobility problems (*unc-1*). The biggest differences are observed in Young Adult stage (YA), therefore experimental results focus on that day.

#### Culture and maintenance of *C. elegans* strains

All worms were maintained in standard dishes measuring 55 mm at 20 as described elsewhere^1^. Wild type animals (i.e. the Bristol N2 strain) were obtained from the Caenorhabditis Genetics Center (CGC, Minneapolis, MN, USA). Uncoordinated animals contain a nonsense mutation, *vlt10*, in the *unc-1* gene that alter locomotion significantly^26^. In this work, we have developed a strain, RVM66, which carries two copies of the transgene *vltIs66[unc-25p::144CAG]; myo-2p::mCherry*. Untranslated CAG expansion produces CAG transcripts that induce dysfunction of GABAergic neurons. The transgene has been inserted into a known site of the *C. elegans* genome using the MosSCI technology^27^. This strain shows a much subtler motor defect compared to *unc-1* mutants. Nematodes were grown in NGM (Nematode Growth Medium) petri dishes and *E. coli* OP50 were used as a food source.

#### Manual motility assay

To evaluate worm motility capacity (i.e. fitness and locomotion-based healthspan) we collected data from a locomotion phenotype, the so-called thrashing assays. Briefly, synchronized L1 worms were incubated in NGM plates until they reached the young adult stage. In this phase, we picked individual animals from the NGM plate and put them into a well of a cell culture 24-well-plate, which was filled with M9 buffer (a physiological solution). After acclimation for 30 s, we counted the number of thrashes (head and tail movement simultaneously) for 1 min manually. We analysed at least 30 animals for each strain and we performed each experiment three times.

#### Automated motility assay

For the automatized locomotion assays, we cultured the animals in NGM plates from L1 to the young adulthood stage. An experiment consists of three plates per condition with about 30 worms each plate to guarantee N almost 30. We replicated the experiment four times. The capture method: we place a Petri plate in the plate support of the device. From this instant on, a macroimage sequence is taken at 1 fps. While the macroimage sequence is taken, one 30-s microimage sequence at 2 fps (60 images) is taken worm by worm. Once the microimage sequences of all the worms have been taken, the macroimage sequence capture is stopped and the current Petri plate is exchanged for another new plate, and so on.

**Figure 6:**
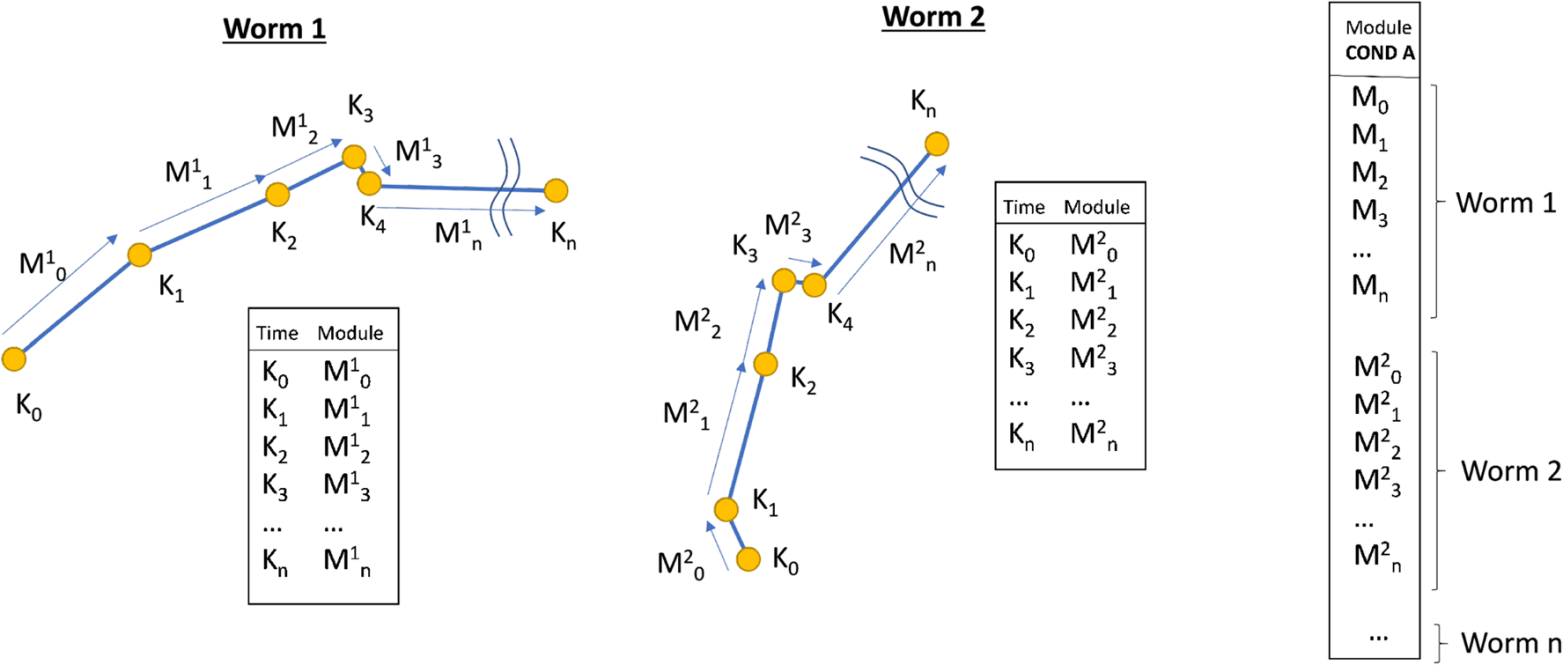
Module index. Every worm movement of every time instant is recorded (worm 1, worm 2 ... worm n).

We calculated several indices, all of them from the control actions (x and y shifting) at each instant *k*, for which the index that gave us significant differences in all cases was one based on the displacement modules, “M”, obtained from Eq. (2), shown in Fig. 6.

2 $$\begin{aligned} M_x=|{\vec{AB}}|= \sqrt{(x_k-x_{k-1})^2+(y_k-y_{k-1})^2}. \end{aligned}$$

#### Statistical methods

The automated and manual methods are different, as stated above. Therefore several statistical methods are used. The data of thrashing index (manual inspection) is parametric, therefore ANOVA test with Tukey’s post-hoc test was used as statistical method for this index. Nevertheless the automated inspection indices are nonparametric data, thus the Wilcoxon rank sum test was utilized. In this case, no statistical correlations can perform it, so results are compared qualitatively. MATLAB R2020b was used as statistical tool.

## Results and discussion

**Figure 7:**
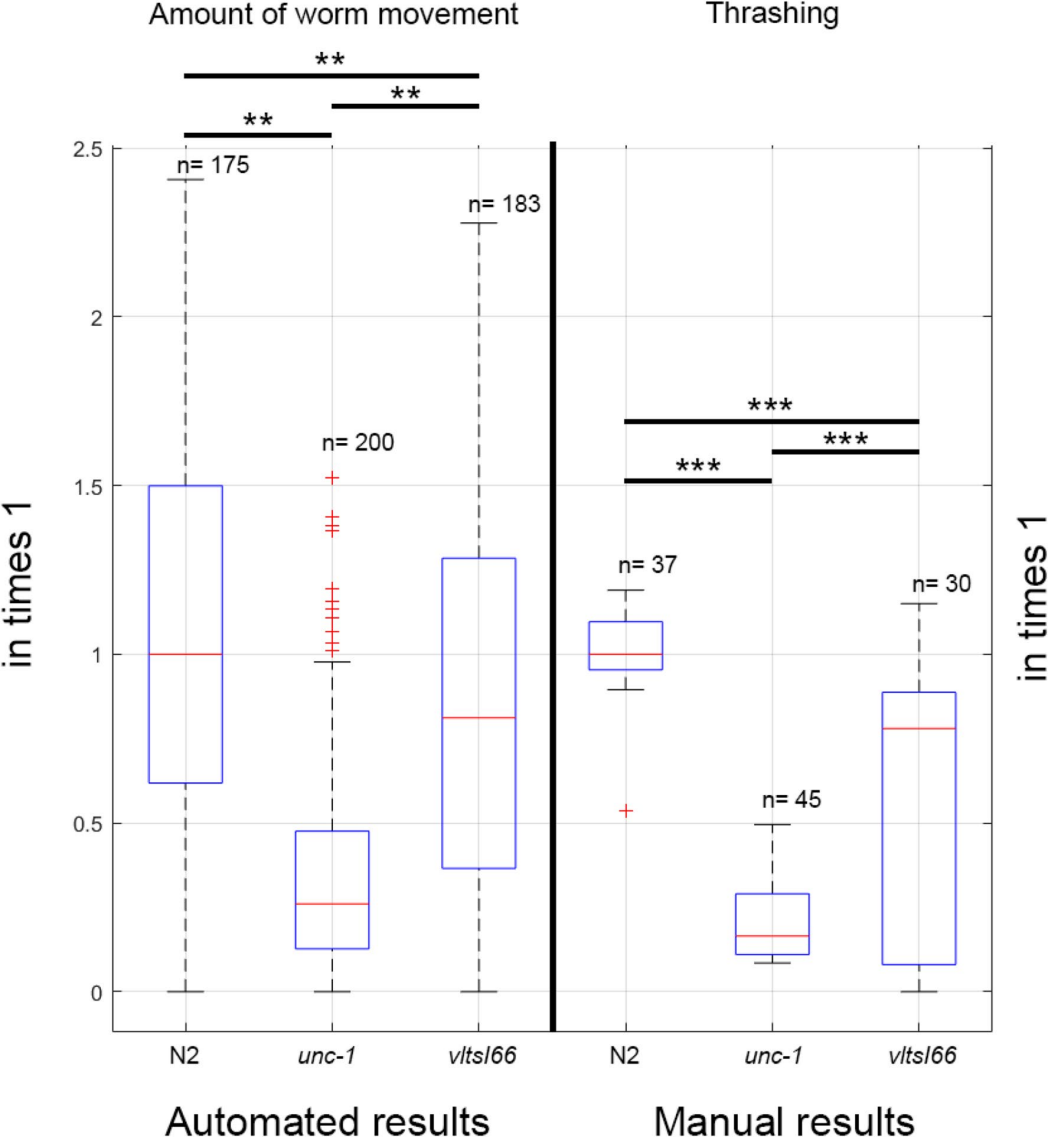
The module index for automated inspection and the thrashing index for manual inspection are shown, which are both transformed into percentages to facilitate visual comparison. On the left is the module sum index. On right, is the graphical output of the manual analysis of the movement capacity. Strains for both experiments are wild type and mutant worms. Wild type (N2 Bristol), *unc-1* mutants and transgenic worms expressing CAG expansions in GABAergic neurons show very different motor capacity in liquid. **Means $p{\text{-value}} < 0.05$, with Wilcoxon rank sum test. ***Means $p{\text{-value}} < 0.001$, in ANOVA test with Tukey’s post-hoc test. The image was drawn with MATLAB R2020b by using the ’boxplot’ function.

In worms, neuronal or muscular defects may be translated into movement dysfunction. Hence, to test our system we used three strains of *C. elegans* that shows no motor phenotype (wild type animals, N2 Bristol), worms showing a very mild phenotype (transgenic worms with slightly impaired GABAergic neurons) and *unc-1* mutants, which are severely impaired for movement. These *unc-1* mutants have altered electrical synapse and produce uncoordinated movement in worms^26,28^. We analysed the motor performance of these strains using the so-called thrashing assays, which consist of counting every movement of the whole body made by each animal while swimming. Our manual analyses showed that *unc-1* mutants have, as expected, a severe motor defect compared to wild type worms Fig. 7. The worms expressing CAG expansions in GABAergic neurons show mild motor phenotype, compared to *unc-1* mutants Fig. 7. This mild phenotype of the transgenic worms is attractive to optimise an automated system of locomotion since, for example, a strain showing subtle phenotypes represents a challenge for drug screening.

### The automated system results

The macroimage allows the tracking of all worms on the Petri plate and also controls the device-head positioning, which is achieved by capturing the blue laser point position on the captured image. However, there is a problem with the laser spot, since its beam splits when passing through the beamsplitter: 50% is transmitted and reaches the Petri dish and the other 50% is reflected. Thus, the macrocamera captures the laser pointer twice in the same position at two different magnifications, because these are at different distances: one laser spot capture is further away, which is reflected on the agar surface (small spot) and another closer one is reflected on the beamsplitter (large spot). On the small image area where the laser spot is projected, the laser intense lighting saturates this small area and makes segmentation difficult. We have minimized this point size and it does not cause problems as long as the mechanical tracking movements are quite fast ($v_{max}=8000 {\text{stp/s}}$ and $a_{max}=25,000 {\text{stp/s}}^2$) and accurate.

**Figure 8:**
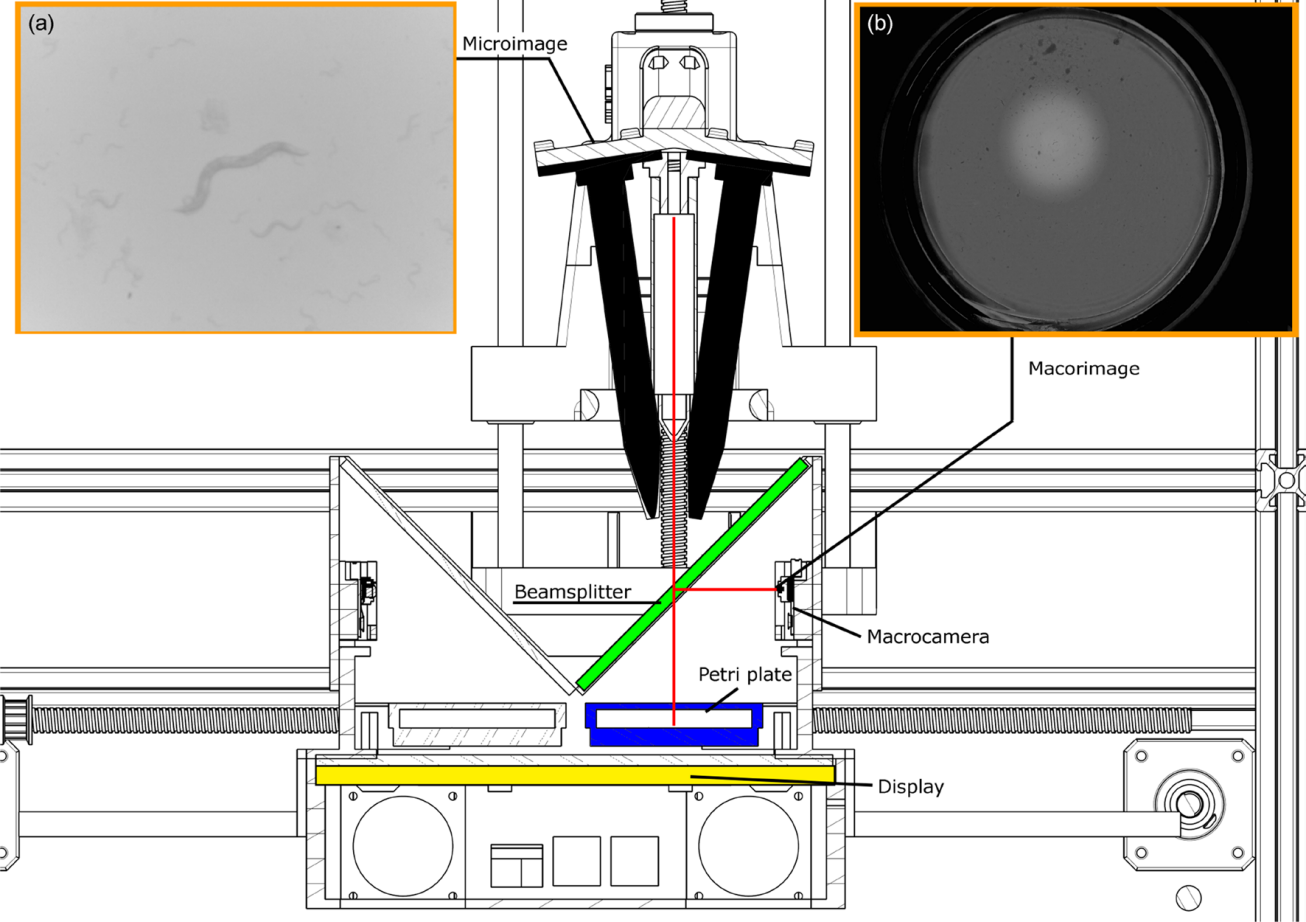
The head and the carriage assembly are shown and how all components interact. The image was obtained with SOLIDWORKS 2020 SP3.0. (**a**) Shows a microimage taken from one of both microcamera. (**b**) Shows macroimage taken from macrocamera.

The luminance control means that the macroimage has the same grey levels throughout the image. In this way, the image quality is higher and the light intensity received by the worms is also controlled^25^. However, microcameras require greater light intensity, for this reason we have implemented a high intensity illumination circle with the centre at the worm-goal position. This circle produces illumination gradients, and thus to solve the image segmentation problem we used two techniques (1) adaptive threshold and (2) a function to generate this lighting circle to soften illumination gradients. This smoothing function is a sigmoidal function that depends on the distance to the circle centre. The result can be seen in the macroimage in Fig. 8a,b. The sigmoid calculation is made at the image grey level in such a way that the controller corrects the image error to obtain a sigmoid with small gradients (Fig. 8, Supplementary Fig. 2).

Tracking is achieved as shown in the Supplementary Video 1, with images captured as shown in Supplementary Fig. 2. The microtracking control actions were recorded in a file. The images of all the plates were taken one by one. In one iteration, the robot can correct the worm positioning while performing microtracking, to keep it centred in both microcamera images.

**Figure 9:**
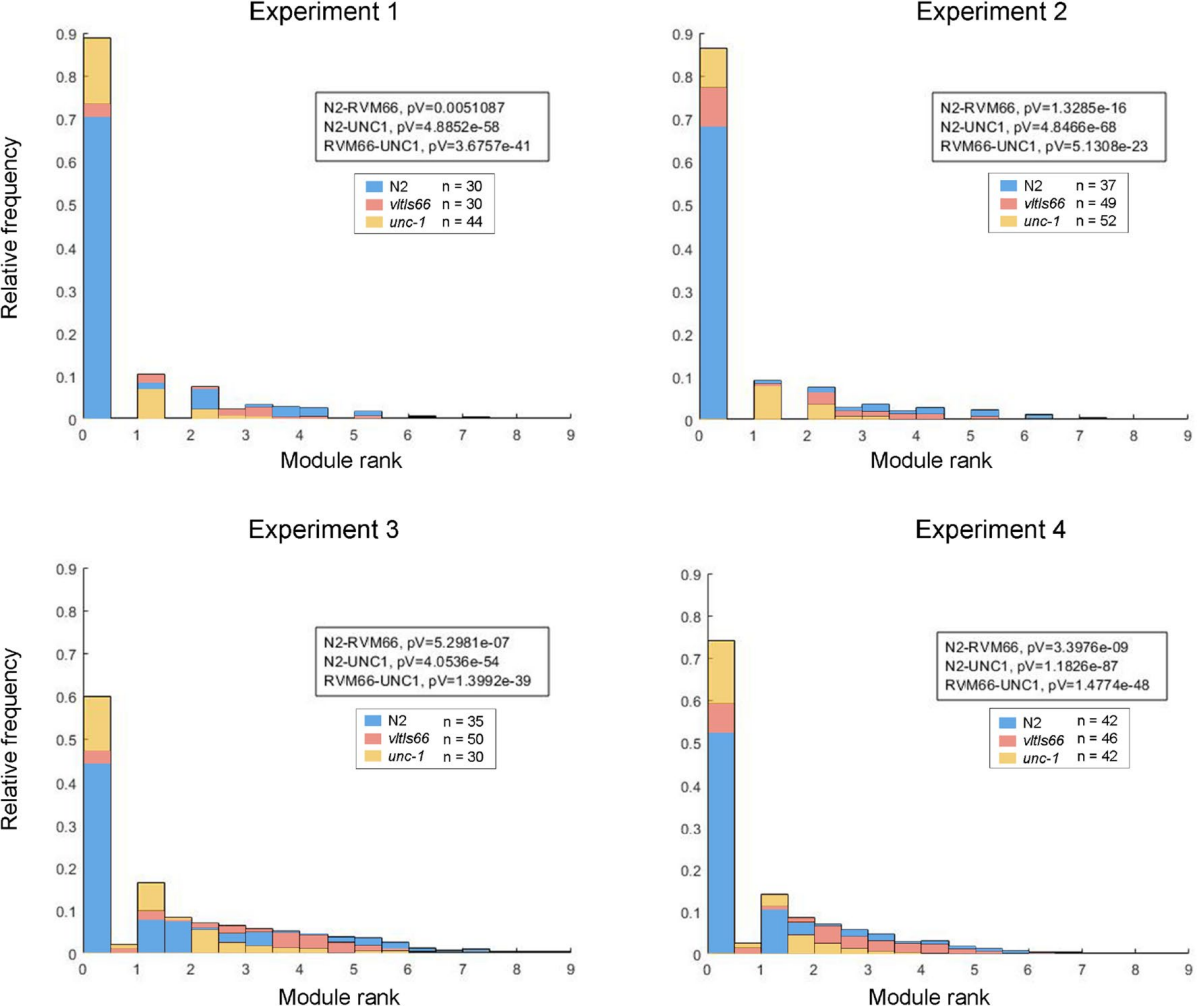
Relative frequency of module values. The abscissa axis represents the module value rank. The units are in steps, which in millimetres are $1.09\, \upmu \mathrm{{m}}/step$. The blue bars are Wild Type (N2) strain, the orange bar is *vltIs66[unc-25p::144CAG]* strains and the red one is *unc-1(vlt10)* strain. The plot was drawn with the ’bar’ function of MATLAB R2020b.

The nematodes were young adults, for which the $p{\text{-value}} < 0.05$ was obtained for all four experiments with the Wilcoxon rank sum test. The most repetitive value rank is the [0-1] rank, which means the worm is most probably static, but this probability depends on strain because some strains demonstrate more movement than others. Figure 9 shows that the wild type (N2 Bristol) has higher mobility, there are greater frequencies of higher movement modules, that is, there is more probability of longer movements. This is confirmed, observing the relative frequency of minimum module rank [0-1], which is lower than the other strains. The *unc-1(vlt10)* mutant is markedly different, where all the module frequencies are closer to 0, which obviously means it has shorter movements. While the *vltIs66[unc-25p::144CAG]* mutant closely resembles the wild-type strain N2, although slightly smaller relative frequencies of the modules than the wild type can be observed. p-values demonstrate figure observations; thus, we can affirm *vltIs66[unc-25p::144CAG]* has a statistically significant reduction in mobility. The module index is transformed in Fig. 7 to allow visual comparison with thrashing method. The module transformation index consists of the module sum for each worm, which provides the amount of movement per worm, and this is expressed in percentage.

We also performed experiments with 1-day-old adult worms, but as with the manual study, on day 1 the differences are minimized, and the statistics are not significant. However, this method opens new paths towards achieving analysis that can provide more information than those obtained to date.

The proposed automated method allows extracting different features to quantify worm movement. As a proof-of-concept, the indices proposed are simple and not equal to the manual method of counting trashing events within a certain time period. In the future other types of movements (e.g., body bend, forward and backward movements) that can be more easily replicated between the two approaches, will be considered.

Although the differences in behaviour between conditions and/or strains are very subtle, statistical differences could be found with our method. This automated method provides similar results than the manuals, besides keeps worm identity with low-resolution camera and tracking worms in high-resolution camera, what allows worms move in a less restricted space. This differs from the rest of the automated methods, and it is important to analyse these behaviours in greater detail. In this way, all worms can be assessed individually at two different resolutions, in a less restricted setting, with longer and more coordinated movements. This allows for more natural social interactions of multiple worms in a less artificial environment.

Contact and interaction with other worms may cause the tracking algorithm to lose the identity of the tracked animal. If the tracked worm identity is lost, some acquired data may require censorship. In our case, where approximately 30 worms were contained in a standard Petri dish measuring 55 mm in diameter, the probability of contact between them is approximately 3%^29^.

If there were a greater number of worms in the Petri dish, the probability of contact and interaction between worms would increase. As a future work, it would be interesting to solve the problem of re-identification of worms using the high-resolution images by trying to avoid data cancelation in a future study.

In addition, future work could integrate other locomotion behaviors, such as body bend, forward movement, backward movement and others. Furthermore, this method has paved the way to solving motion pattern classification problems and other assays using high-resolution images.

## Conclusions

We have demonstrated the multi-view Cartesian robot can inspect *C. elegans* in standard Petri dishes and extract information to find statistical differences between *C. elegans* movements, with a simple index based on the position variation modules in each sampling period, k. These differences can be observed between strains with very subtle differences (wild type and *vltIs66[unc-25p::144CAG]*). Multi-view monitoring provides greater data-collection flexibility because it offers more than one resolution, which allows all worms to be tracked at once, while also tracking one animal at high-resolution. This method can also monitor worm movement in standard Petri dishes (55 mm in diameter), which improves negative behavioural alterations caused by smaller spaces.

New image processing studies will be made possible given this increased amount of available data, facilitating the analysis of *C. elegans* movement characteristics. Possibly, accumulative data collection will shed light on characteristics in the physiognomy, movement or posture of the nematodes that define characteristic group behaviour, which other analyses cannot detect.

This method can also capture the image with two microcameras, thus future works on 3D reconstructions can be made of each worm in the Petri dish.

## Supplementary Information

Supplementary Figures.

Supplementary Video 1.

## Data Availability

To include, in this order: Accession codes (where applicable).
