# Supplementary material for: Multiview motion tracking based on a cartesian robot to monitor Caenorhabditis elegans in standard Petri dishes
Source: Sci Rep. 2022 Feb 2;12:1767. doi: 10.1038/s41598-022-05823-6 (PMC8810772; doi:10.1038/s41598-022-05823-6)
Supplement: Supplementary file 1 — Supplementary Figures. [file 41598_2022_5823_MOESM1_ESM.pdf]

Multiview motion tracking based on a cartesian robot to monitor *Caenorhabditis elegans* in standard Petri dishes

Joan Carles Puchalt[1], Jose F. Gonzalez-Rojo[1], Ana Pilar Gómez-Escribano[2], Rafael P. Vázquez-Manrique[2], and Antonio-José Sánchez-Salmerón[1,*]

[1]Universitat Politècnica de Valéncia, Instituto de Automática e Informática Industrial, Valencia, Spain
[2]Laboratory of Molecular, Cellular and Genomic Biomedicine, Instituto de Investigación Sanitaria La Fe, Valencia, Spain and Centro de Investigación Biomédica en Red de Enfermedades Raras (CIBERER), Valencia, Spain
[*]asanchez@isa.upv.es

Contents

Worm detection algorithm 3

Worm tracked........ 4

Tracking procedure 5

Worm detection algorithm

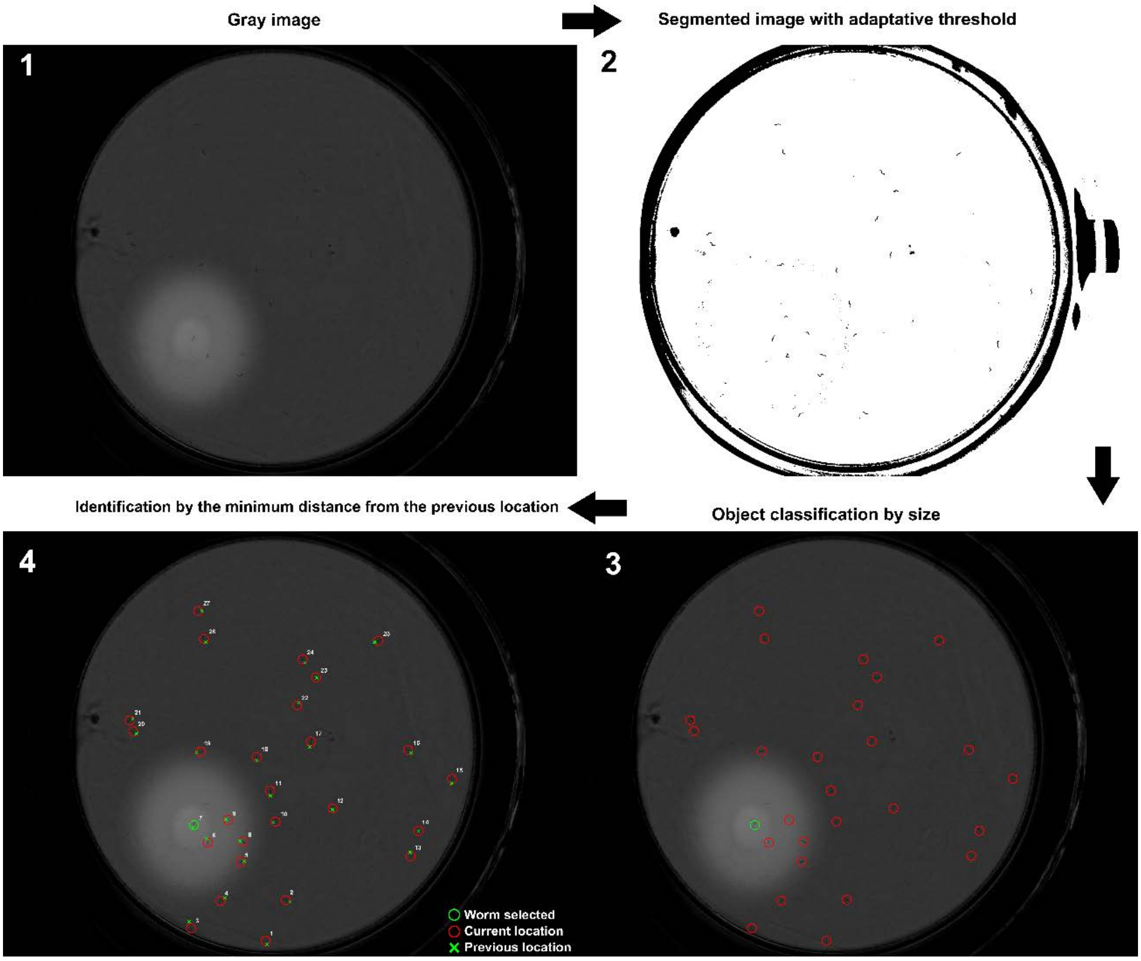

Supplementary Figure S1. Worm detection algorithm. The graph is obtained with OpenCV. The gray image (1) is segmented with adaptative threshold (2). The detected objects are classified by size (3). And (4) identification is made by the minimum distance from previous location.

Worm tracked

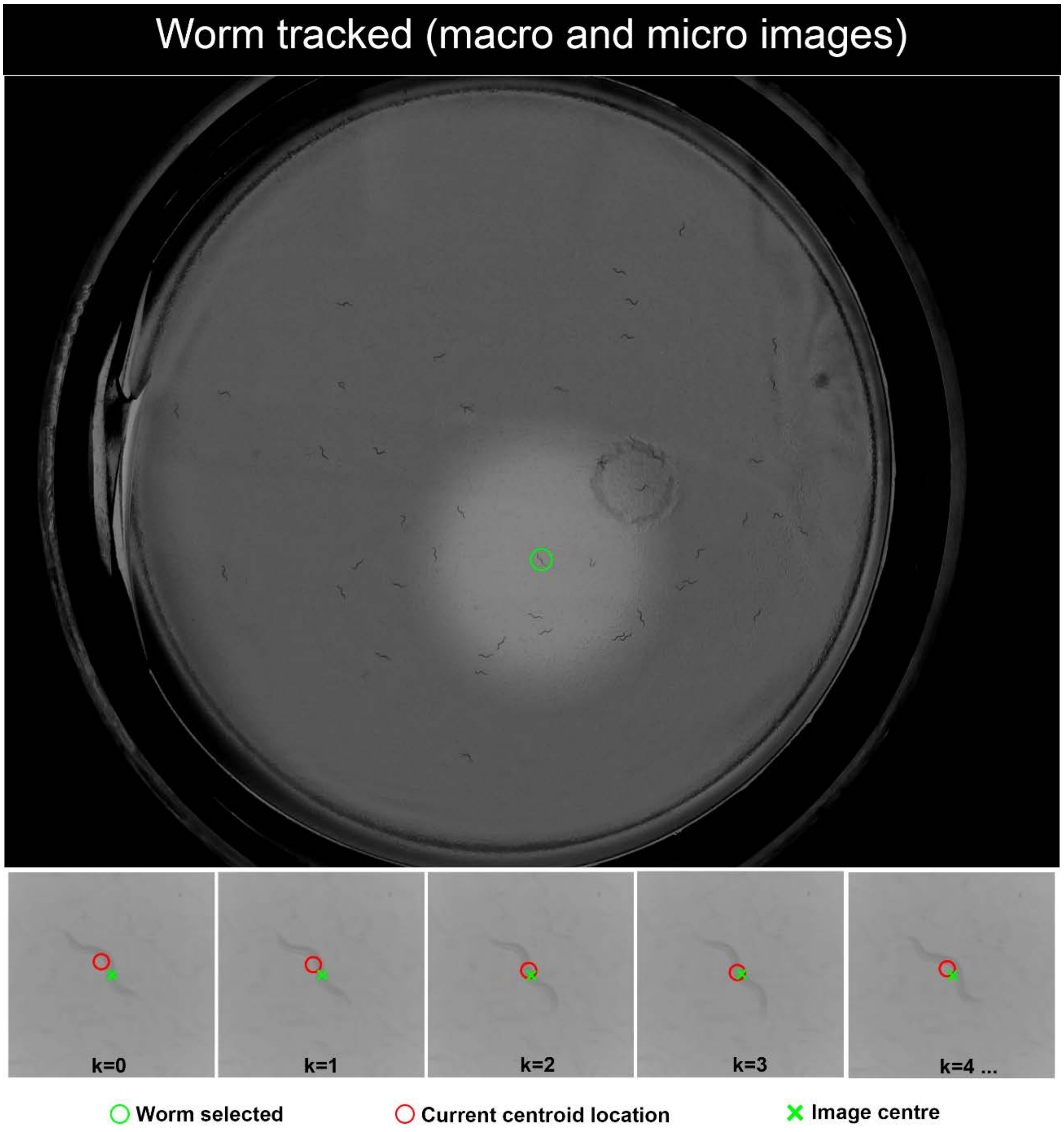

Supplementary Figure S2. Worm tracked. The graph is obtained with OpenCV. When a worm is selected, a backlight area is illuminated to maximum in order to increase light for microcameras. In this figure can be seen the image quality obtained by both cameras while makes tracking.

Tracking procedure

Supplementary Video 1. Tracking procedure. First, a worm is selected in the macroimage sequence. Second, laser is switched on. Third, difference between target worm and laser location is measured. Fourth, control action is applied. Fifth, when worm is reached, starts the microimage sequence. Sixth, microimage is focused in. And finally microtracking is made, while image sequences are captured for a time.
